# Introduction of a novel magnetic resonance imaging-based scoring system for assessing disease activity in children with juvenile dermatomyositis

**DOI:** 10.1093/rheumatology/key144

**Published:** 2018-06-12

**Authors:** Mandela Thyoka, Oba Adekunle, Clarissa Pilkington, Stephen Walters, Owen J Arthurs, Paul Humphries, Karl Johnson, Jeannette Kraft, Caren Landes, Thara Persaud, Raj Sinha, Amaka C Offiah

**Affiliations:** 1 Department of Radiology, Sheffield Teaching Hospitals, Sheffield, UK; 2 Department of Paediatric Rheumatology, Great Ormond Street Hospital for Children NHS Trust, London, UK; 3 School of Health & Related Research, University of Sheffield, Sheffield, UK; 4 Department of Radiology, Great Ormond Street Hospital for Children NHS Trust, London, UK; 5 Department of Radiology, University College London, London, UK; 6 Department of Radiology, Birmingham Children’s Hospital, Birmingham, UK; 7 Department of Radiology, Leeds Teaching Hospitals NHS Trust, Leeds, UK; 8 Department of Radiology, Alder Hey Children’s NHS Foundation Trust, Liverpool, UK; 9 Department of Radiology, Royal Manchester Children’s Hospital, Manchester, UK; 10 Department of Radiology, Newcastle Upon Tyne Hospitals NHS Foundation Trust, Newcastle, UK; 11 The University of Sheffield, Academic Unit of Child Health, Sheffield Children’s NHS Foundation Trust, Sheffield, UK

**Keywords:** juvenile dermatomyositis, magnetic resonance imaging (MRI), scoring system, children, creatine kinase, electromyogram

## Abstract

**Objectives:**

We aimed to develop and assess the reliability of a novel MRI-based scoring system for reporting the severity of MRI findings in children with suspected JDM.

**Methods:**

Nine consultant paediatric radiologists independently assessed and scored 40 axial and 30 coronal thigh MR images of children with suspected JDM on two occasions using the juvenile dermatomyositis magnetic resonance Imaging Score (JIS). JIS was calculated for both reads for each plane and each limb, with possible scores ranging from 0 (normal) to 100 (severe). Inter- and intraobserver agreement was calculated using the intraclass correlation coefficient (ICC) and two- and one-way random effects models, respectively. Bland-Altman plots of the difference in JIS against the average JIS were also produced for each rater.

**Results:**

Overall, the interobserver reliability and agreement was good—for axial images, JIS ranged from 46.8 to 61.0 [ICC = 0.88 (95% CI: 0.82, 0.92)] for the left limb and 47.9–61.4 [ICC = 0.87 (95% CI: 0.81, 0.92)] for the right limb. For coronal images, JIS ranged from 56.7 to 65.1 [ICC = 0.90 (95% CI: 0.85, 0.95)] for the left limb and 55.7 to 66.8 [ICC = 0.90 (95% CI: 0.84, 0.94)] for the right limb. The intraobserver reliability and agreement was good, with ICC ranging from 0.90 to 0.94.

**Conclusion:**

JIS is a semi-objective scoring system with potential to serve as a reliable biomarker of disease severity and response to therapeutic interventions in children with JDM.

**Rheumatology key messages**

- We have developed a consensus MRI protocol for JDM.
- We have developed a reliable MRI-based semi-quantitative scoring tool for JDM (JDM MRI Score).
- JDM MRI Score provides a standardized tool for clinical and research use.

## Introduction

JDM is a rare autoimmune disease in children (<16 years of age). It is a systemic vasculopathy, characterized by proximal muscle weakness, raised muscle enzymes and characteristic skin rash (Gottron’s papules of extensor surfaces and a heliotrope rash of the eyelids). JDM is the most common inflammatory myopathy in childhood [1], with an estimated annual incidence of 2–3/million children. The median age of onset is ∼7 years. Girls are more commonly affected than boys [2, 3].

In 1975 Bohan and Peter [4, 5] published criteria for the diagnosis of JDM. Definite diagnosis of JDM is made in the presence of the characteristic skin rash plus three or of four of the following: systemic proximal muscle weakness; elevated muscle enzymes; characteristic pathology on muscle biopsy; and a myopathic picture on EMG. The combination of the rash plus three or four criteria confirms the diagnosis; the rash plus two criteria indicates probable disease, while the rash plus one criterion indicates possible disease. To date this remains the only validated diagnostic system, despite the fact that it predates widespread clinical use of MRI and therefore does not include MRI as one of the diagnostic criteria.

MRI is an imaging modality that does not expose the child to ionizing radiation. The study by Kaufman *et al.* [6] was one of the earliest to assess the role of MRI for JDM (and polymyositis). In the context of JDM, i.v. contrast administration is probably not indicated, and because affected children are usually of a co-operative age, sedation is seldom required. As such, MRI in suspected JDM is relatively non-invasive (less so than muscle enzymes, EMG and muscle biopsy for example). MRI has been used both for the diagnosis of JDM (including selection of sites for muscle biopsy) and to monitor treatment [1, 7–9]. Diagnostic sensitivity of MRI is 76% compared with 64% for elevated serum creatine kinase [4]. Furthermore, increased MR signal intensity secondary to inflammation within muscle correlates better with disease activity than does elevated creatine kinase [7, 10, 11]. Finally, MRI was considered an important diagnostic criterion and (together with muscle biopsy) rated the most useful clinically relevant investigative criterion for JDM by 78 respondents of a survey of members of the Network for JDM and the Paediatric Rheumatology International Trials organization [12].

Given that JDM is associated with a proximal myopathy, it is generally accepted that images of the gluteal and thigh muscles are sufficient for both diagnosis and follow-up. In the UK the practice is varied with MRI images obtained in the coronal plane alone, in the axial plane alone or (rarely) in both planes depending on local preference. However, some European centres are performing whole-body MRI in suspected JDM [13]. To date, no objective assessment has been made as to which plane or body site is most reliable for assessing disease activity in JDM. Similarly, there is variation between centres regarding the precise sequences to perform. Various combinations of techniques—T1-weighted (muscle atrophy; chronic disease) and T2-weighted/fat suppression (soft tissue oedema; active disease)—provide useful information [7, 10, 11, 14]. Short tau inversion recovery (STIR) sequences improve the visualization of inflammatory change in the skin, subcutaneous tissues and fascia, which are often undetected by standard T1 and T2 sequences [14].

Muscle T2-weighted relaxation times differ significantly in children with active JDM compared with both children with inactive JDM and healthy children, and T2-weighted relaxation times could therefore serve as a quantitative measure of muscle inflammation [7, 8]. However, measurement of T2-weighted relaxation times requires computer software that is not widely available and operator-dependent selection of regions of interest (which is difficult in the presence of muscle wasting), and does not assess other parameters such as subcutaneous inflammation, perifascicular oedema, calcinosis and muscle atrophy. Uniformity in performing and reporting MR images is necessary from a clinical point of view, and if improvements are to be made in health outcomes for affected children, then reliable imaging and reporting are also important in the research setting; MRI has the potential to be used as a relatively rapid, non-invasive and reliable endpoint for clinical trials. In summary, despite widespread utility, there is currently no uniformity in the MRI planes and sequences or in the reporting of MRI scans for suspected or confirmed JDM.

We have previously developed and published an MRI-based scoring system for JDM [15]. Although intraobserver reliability was good, interobserver reliability was only fair. Considering the latter limitation, the purpose of this current study was to refine the scoring system including the involvement of a larger panel of observers, in order to improve reliability prior to a prospective validation study. As a secondary goal, we sought to determine which MRI sequences and planes should routinely be performed when JDM is suspected so as to provide uniformity between practitioners.

## Methods

### Panel of assessors

Nine UK-based paediatric radiologists with an interest in musculoskeletal imaging constituted the multicentre reader panel. The panel had two roles: collective modification of the scoring system and independent assignment of scores to pre-selected, anonymized MRI scans of thighs for suspected JDM.

### Development of the scoring system

The accuracy of the previously proposed scoring system was acceptable for the single reader but showed high variability between two different individuals [15]. In order to address this issue, nine paediatric radiologists and two paediatric rheumatologists (Liza McCann and C.P.) convened in January 2014 for a preliminary roundtable meeting (UK JDM Imaging Group, chaired by A.C.O.). During this meeting, discussions were held regarding developing a protocol for the MRI planes and sequences in addition to optimizing the scoring system. The panel discussed the previous MRI scoring system [15], identifying its limitations. It was felt that the interobserver variability of the previous system was largely due to the relative subjectivity of defining degree of inflammation for whole muscle groups. Therefore, rather than modifying the system, the group developed and familiarized themselves with a novel MRI scoring system—JDM Image Score (JIS), using a set of MR images from 20 patients. These images, constituting the familiarization set, did not feature further in the project.

### Selection of study MR images

The MRI protocols for suspected JDM at the institutions of panel members at the time of the study are summarized in the supplementary Table S1, available at *Rheumatology* online. MRI studies were selected and anonymized by each panel member before being sent to O.A. to collate, with a total of 50 axial and 32 coronal studies received. Of these, 40 axial and 30 coronal studies were selected to cover the spectrum of disease severity. The image selection and mailing procedure was developed to reduce image recall by observers and any effects of a learning curve on assignment of JIS. Panel members read the digital imaging and communications in medicine images from the compact discs on high-resolution diagnostic monitors in identical National Health Service settings to those used for interpretation of images during usual clinical practice. Panel members had full access to image manipulation (magnification, brightness, etc.), used at the observers’ discretion.

### JIS

**Fig. 1:**
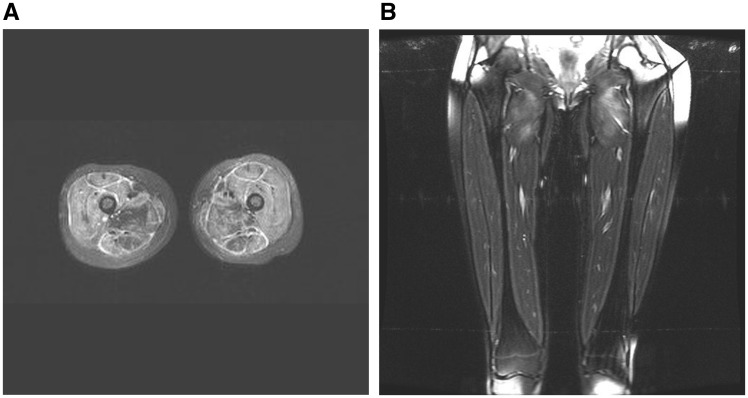
MRI images of a sample case used to develop JIS (**A**) Axial image showing inflammation of all muscle groups and perifascicular oedema. (**B**) Coronal image of another patient, showing inflammation of the proximal adductor muscles only. JIS: Juvenile dermatomyositis magnetic resonance Imaging Score.

The JIS was developed based on characteristic features on MRI of affected limbs. JIS ranges from 0 (normal) to 100 (severe), based on the severity, extent and volume of muscle inflammation and the presence of muscle and perifascicular oedema (see JIS, Table 1). Severity of inflammation was assessed on the single most severely affected slice on the plane being reported according to a categorical scale (0 = none, 2 = mild, 4 = moderate, 6 = severe). The extent of muscle inflammation was assessed by an overall estimation of the number of consecutive slices involved in the superoinferior (axial studies) or anteroposterior (coronal studies) direction. Similarly, the volume of muscle inflammation was assessed by an overall estimation of the relative volume of affected muscle. The presence of perifascicular oedema was weighted with a relatively high score, given the panel’s opinion that children with perifascicular oedema have more severe disease and are more likely to develop calcinosis. Additional assessment, though not forming part of JIS, was made of the presence of muscle atrophy and calcification. Using the thigh of each limb, all four muscle groups (adductors, gluteals, hamstrings and quadriceps) were independently assessed and scored for inflammation. Additionally, the presence of perifascicular oedema was scored (0 = absent, 14 = present). The total scores were calculated to give the JIS. Fig. 1 shows example images that were scored as part of this study.

**Table 1:** JDM MRI score

Muscle group	Muscle inflammation			Oedema		Total
	Severity	Extent	Volume	Soft tissue	Perifascicular	
Adductors						
Gluteals						
Hamstrings						
Quadriceps						
Any location^a^						
Total						

For each plane (axial and coronal) and each limb (left and right) independently score: muscle inflammation: 0 = none, 2 = mild, 4 = moderate, 6 = severe; soft tissue oedema: absent = 0, present = 14; perifascicular oedema: absent = 0, present = 14. Minimum score = 0 (normal); maximum score = 100 (severe). Additionally, please note presence of (i) muscle atrophy and (ii) calcification. ^a^Applies only to oedema. The involved muscle group(s) must be specified for muscle inflammation.

**Fig. 2:**
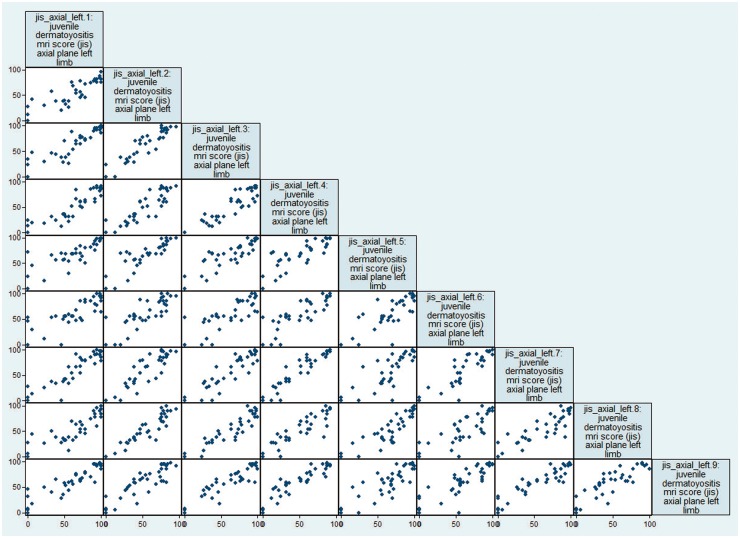
Scatter plot matrix graph of JIS for left limb, axial plane JIS: Juvenile dermatomyositis magnetic resonance Imaging Score.

### Independent assignment of JDM image scores

Panel members independently scored all image sets on a pre-designed online score sheet according to the newly developed JIS (Table 1). For each score, the level of observer confidence in assigning that score (5-point Likert scale) was recorded.

### Statistical analysis

All statistical analyses were made using International Business Machines Corporation (IBM) Statistical Package for the Social Science (SPSS) Statistics for Windows, Version 24.0 (IBM Corp., Armonk, NY, USA). The primary outcome was defined as the reliability (inter- and intraobserver) of JIS. The secondary outcome was a comparison of the reliability of JIS for coronal and axial planes. For the MRI-based scoring system to be clinically useful, we decided *a priori* that a minimum acceptable level of reliability should be an intraclass correlation coefficient (ICC) of 0.70 [16, 17].

#### Interobserver reliability

For each individual radiologist, JIS were graphically displayed and their overall mean score calculated. Agreement between the observers (interobserver reliability) using the JIS scoring system was assessed by calculating the ICC; a value of 1.00 corresponding to perfect agreement. The ICC and its associated CI was estimated in SPSS using a two-way random effects model and the absolute agreement method.

#### Intraobserver reliability

For each individual radiologist, a Bland-Altman plot of the mean score of the two reads against the difference between the two reads was produced and complimented by the calculation of the 95% CI for the mean difference. Agreement within the observers (intraobserver reliability) using the JIS scoring system was assessed by calculating the ICC; a value of 1.00 corresponding to perfect agreement between the observers for their first and second readings. The ICC and its associated CI was estimated in SPSS using a one-way random effects model and the absolute agreement method. Bland-Altman plots of the difference in JIS (first to second reading) against the average JIS (first + second reading) were also produced for each rater and the paired difference and its associated 95% CI calculated from a paired *t*-test.

### Ethics approval

Formal Research and Ethics Committee approval was not required for this study, which involved retrospective review of anonymized images. The central institution Sheffield Children's Hospital NHS Trust approved the protocol, confirming that Research and Ethics Committee approval was not required, and provided indemnity (R&D: SCH/13/057).

## Results

Nine of the 10 raters from eight UK National Health Service Trusts (supplementary Table S1, available at *Rheumatology* online) completed the full task, using JIS to score axial and coronal images, left and right sides, on two occasions. The raters were consultant paediatric radiologists with a mean of 9.6 (range 4–18) years’ experience between them. All but one of the eight centres used both axial and coronal planes in their MRI protocol (one centre used the axial plane only) and the sequence common to all centres was the STIR (supplementary Table S1, available at *Rheumatology* online). Fig. 2 shows an example scatterplot matrix graph of individual scores (JIS) of the nine raters scoring one limb (left) in one plane (axial) only.

### Interobserver reliability

**Fig. 3:**
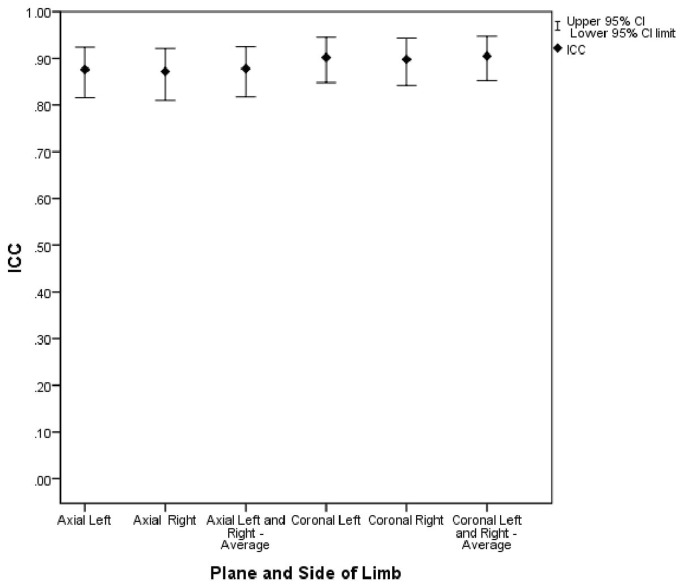
ICC (with 95% CI) for axial and coronal planes (ICC = 0.0–1.0) ICC: intraclass correlation coefficient; JIS: Juvenile dermatomyositis magnetic resonance Imaging Score.

Overall, interobserver reliability and agreement between the nine raters was good (Fig. 3). For the 40 axial images, the mean JIS for the left limb, across the nine raters ranged from 46.8 to 61.0 and the ICC was 0.88 (95% CI: 0.82, 0.92), and for the right limb the mean JIS, across the nine raters, ranged from 47.9 to 61.4 and the ICC was 0.87 (95% CI: 0.81, 0.92).

For the 30 coronal images the mean JIS for the left limb, across the nine raters, ranged from 56.7 to 65.1 and the ICC was 0.90 (95% CI: 0.85 to 0.95), and for the right limb the mean JIS, across the nine raters ranged from 55.7 to 66.8 with an ICC of 0.90 (95% CI: 0.84 to 0.94). The intraobserver reliability and agreement for JIS was also good, with the ICC values between the first and second reads ranging from 0.90 to 0.94 (Table 2).

**Table 2:** Intraobserver reliability of the nine observers (test–re-test) for coronal and axial images of left and right limbs

			Mean JIS		Paired difference (95% CI)	ICC (95% CI)
Plane	Limb	N	First reading	Second reading		
Coronal	Left	54	58.3	59.4	−1.1 (−4.7, 2.4)	0.91 (0.85, 0.95)
Coronal	Right	54	58.7	60.2	−1.6 (−5.3, 2.1)	0.90 (0.84, 0.94)
Axial	Left	72	56.1	56.4	−0.3 (−3.2, 2.5)	0.93 (0.89, 0.96)
Axial	Right	72	57.0	56.7	0.3 (−2.3, 2.9)	0.94 (0.91, 0.96)

The nine raters each re-scored six different coronal images a second time, and re-scored eight different axial plane images a second time. JIS: Juvenile dermatomyositis magnetic resonance Imaging Score.

## Discussion

Our study shows that JIS has good intra- and interobserver agreement among paediatric radiologists when used to assess the degree of muscle inflammation in suspected cases of JDM and that the scores for disease severity are the same in either axial or coronal planes. The level of reliability of JIS is well above the minimum level of clinical usefulness and applicability (0.70) [16, 17]. Similarly, we have shown JIS to be independent of the plane of imaging, with similar observer reliability whether reporting from axial or coronal images. The previous scoring system [14] assessed extent of muscle inflammation considering the entire muscle group under interrogation. This is likely to have led to the fair interobserver reliability. In contrast, JIS considers severity, extent and volume of muscle inflammation, providing the observer with narrower parameters by which to score the scans and resulting in a higher interobserver reliability. These results imply that JIS can be used both as a reliable and semi-objective tool for the diagnosis and monitoring of JDM in clinical practice and as a reliable biomarker for disease severity in research trials.

Although there have been a few attempts to develop diagnostic criteria/classification systems for both adult and juvenile idiopathic inflammatory myopathies [18–21], as far as we are aware, to date the only validated diagnostic system in use specifically for JDM does not include MRI findings [4, 5], despite the fact that MRI plays a fundamental role in the clinical management of JDM, both as a diagnostic tool and to monitor response to treatment [22].

The major limitation to the use of MRI is the lack of uniformity in both the reporting/interpretation of MRI findings in the clinical setting and in the planes and sequences performed; imaging protocols are based on local preferences with some centres only obtaining images in a single orthogonal plane (coronal or axial) while others use both planes. This lack of uniformity may partially explain why MRI (despite widespread availability, lack of invasiveness and relatively rapid results) is not a diagnostic criterion for JDM. Recently, modifications to the current diagnostic criteria have been proposed to include evidence of myopathy on MRI [12, 23]; as such, JIS could prove invaluable in ensuring comparable, uniform reporting and interpretation.

We assessed MR images of the gluteal and thigh muscles for JIS as these sufficiently locate the proximal myopathy that is a clinical feature of JDM. Although no objective assessment was made as to which plane is the most useful in terms of correlation with clinical severity, our study has shown no significant difference in MRI severity scores when using either the axial or coronal planes. Various combinations of techniques—T1-weighted (muscle atrophy; chronic disease) and T2-weighted/fat suppression (soft tissue oedema; active disease)—provide useful information. STIR sequences improve the visualization of inflammatory change in the skin, subcutaneous tissues and fascia, which are often undetected by standard sequences. There is nothing to be gained by performing both a T2-weighted and an inversion recovery sequence. Others have advocated the use of whole-body MRI as it provides additional information to clinical evaluation in estimation of total inflammatory burden when compared with clinical assessment of disease activity by such measures as the Manual Muscle Test and the Childhood Myositis Assessment Scale [13]. Although whole-body MRI may be a reliable tool in assessing the full extent of the disease, we advocate the use of MRI of proximal lower limb muscles as this is the site of predilection of the inflammatory changes in JDM and because localized MRI is now available in most paediatric centres, whereas whole-body MRI is not. As far as we know, no formal studies have been performed to assess the role of contrast in the context of suspected JDM; nevertheless, the panel agreed that i.v. gadolinium was not needed, even though some of the centres had until this study been routinely using it.

An MRI-based scoring system such as JIS offers significant advantages in comparison with other diagnostic investigations (muscle enzymes, EMG or muscle biopsy) that are currently used in children with suspected JDM. Firstly, MRI is a relatively non-invasive, non-ionizing investigation and neither contrast administration nor sedation is usually required in the setting of suspected JDM. In addition, MRI can be used to guide biopsies when JDM is suspected [24]. The diagnostic sensitivity of MRI compares favourably to that of elevated serum creatine kinase and the increased signal intensity seen on MRI due to muscle inflammation correlates better with disease activity than does elevated creatine kinase [10, 11, 25]. MRI discriminates between affected and non-affected muscle groups by visualizing oedema from acute inflammation and the signal intensity is associated with disease activity in the acute presentation of JDM. Finally, if local expertise does not extend to reporting of the images, with current advances in information and technology, their transfer across centres allows ease of access to MRI images by specialist radiologists. If the correct plane and sequences are obtained locally, this will significantly speed up the diagnostic pathway for the patient at the specialist referral centre and allow prompt initiation of appropriate therapy and improved patient outcomes.

There are several limitations of this study. Firstly, as we did not have access to the clinical data to provide an index of clinical disease assessment, we were not able to show the difference in the clinical value of either the axial or coronal sets of images. However, our study has shown that there is agreement between these two orthogonal planes when scoring for radiological disease severity markers and therefore images are required in only one plane. Secondly, the study is limited by its retrospective design; however, our chosen methodology is appropriate for the purposes of developing a scoring system. Thirdly, since it is known that the intensity of inflammation on MR scans decreases significantly after treatment, but the histologically detected inflammation does not change substantially [20], an MRI-based score may be beneficial at the onset prior to commencement of treatment, but the score may underestimate the burden of inflammation once treatment has commenced. Fourthly, JIS is a semi-objective scoring system, which means some observer subjectivity cannot be avoided; however, we could further have reduced subjectivity by providing guidelines for defining muscle involvement, for example, volume (mild ⩽25%, moderate = 26–50% and severe ⩾1% involvement). The use of such criteria should be assessed in a future prospective study. Furthermore, we gave perifascicular oedema a weighted score because of its prognostic importance as, according to our panel, children with perifascicular oedema have more severe disease and are more likely to develop calcinosis. The weighted score of 14 was chosen to allow JIS to reach a convenient maximum score of 100. This may overemphasize the significance of perifascicular oedema but will do so equally in all affected patients. Finally, JIS has not been clinically validated in a multicentre prospective study—this is our intended next step and the prospective nature of such a study will also allow longitudinal comparison of JIS and other clinical and laboratory parameters of disease progression.

In conclusion, JIS is a reliable semi-objective method of conveying the degree of muscle inflammation in suspected JDM and has good intra- and interobserver agreement among paediatric radiologists in both axial and coronal planes. We recommend routine axial STIR **(**or T2 weighted fat saturated) and T1 weighted sequences of the thighs as the disease severity scores are the same in either axial or coronal planes. JIS has potential to provide clinicians and researchers with a reliable biomarker of disease severity and response to therapeutic interventions in children with JDM and as such provide a uniform platform for reporting clinical and research findings.

## Supplementary Material

Supplementary Table S1
