# Supplementary material for: Introduction of a novel magnetic resonance imaging-based scoring system for assessing disease activity in children with juvenile dermatomyositis
Source: Rheumatology (Oxford). 2018 Jun 12;57(9):1661–8. doi: 10.1093/rheumatology/key144 (PMC6105921; doi:10.1093/rheumatology/key144)
Supplement: Supplementary Table S1 [file key144_suppl_table_s1.docx]

**SUPPLEMENTARY DATA**

**Supplementary Table S1: MR protocol for JDM at each center^a^**

| Centre | Scanner | Planes | Sequences |
| --- | --- | --- | --- |
| 1 | 1.5T (GE 1.5T)  1.5T (GE 1.5T) | Axial & coronal  Axial | T1^b^  T2 STIR, T2 FS^c^ |
| 2 | 1.5T (Siemens) | Axial & coronal | T2 STIR, T1 non FS |
| 3 | 1.5T (Siemens & GE) | Axial & coronal | T1, T2, STIR |
| 4 | 1.5T (Siemens) | Axial & coronal | T1, STIR |
| 5 | 1.5T/3T (Philips Ingenia) | Axial & coronal | T1, STIR |
| 6 | 1.5T (Siemens) | Axial & coronal | T1, STIR |
| 7 | 1.5T (Siemens) | Axial & coronal | T1, STIR |
| 8 | 1.5T (Avanto) | Axial | T1, T2 FS, Gad T1 FS |

**^a^**From iliac crests to distal femoral condyles. ^b^Prior to 2010, ^c^after 2010. equencesged from 55.7 to 66.8 wgerom 56.7 to 65.1 iut the upper age limit that defines children.Center codes: 1 Sheffield Children's NHS Foundation Trust, Sheffield, UK; 2 Great Ormond Street Hospital for Children NHS Trust, London, UK; 3 Birmingham Children’s Hospital, Birmingham, UK; 4 Leeds Teaching Hospitals NHS Trust, Leeds, UK; 5 Alder Hey Children's NHS Foundation Trust, Liverpool, UK; 6 Royal Manchester Children's Hospital, Manchester, UK; 7 Newcastle Upon Tyne Hospitals NHS Foundation Trust, Newcastle, UK; 8 University College Hospital NHS Trust, London, UK. STIR: Short tau inversion recovery; FS: Fat suppression; Gad: Gadolinium.
